# Effects of Environmental Quality Perception on Depression: Subjective Social Class as a Mediator

**DOI:** 10.3390/ijerph18116130

**Published:** 2021-06-06

**Authors:** Liqin Zhang, Lin Wu

**Affiliations:** 1 School of Sociology, Wuhan University, Wuhan 430072, China; zhangliqin-psy@shzu.edu.cn; 2 Department of Psychology, Shihezi University, Shihezi 832000, China; 3 Psychological Application Research Center, Shihezi University, Shihezi 832000, China

**Keywords:** environmental quality perception, living environmental quality perception, subjective social class, depression

## Abstract

Although the relationship between environment and public depression has aroused heated debate, the empirical research on the relationship between environmental quality perception and public depression is still relatively insufficient. This paper aims to explore the influence of environmental quality perception on public depression and the mediating role of subjective social class between environmental quality perception and public depression. Using the China Family Panel Studies data of 2016 for empirical analysis, this study’s results show that environmental quality perception has a significant effect on public depression and subjective social class also has a significant effect on public depression. In addition, we found that subjective social class can play a partial mediating role between environmental quality perception and public depression, and the intermediary effect only comes from the contribution of the perception of living environmental quality, not the perception of overall environmental quality. That is to say, the perception of living environment quality deeply affects the subjective social class, and then induces public depression. In order to alleviate the relationship between environmental quality and public depression, it is recommended that the state environmental protection department and civil affairs department strengthen the improvement of public living environment so as to promote individual subjective social class and reduce the risk of public depression. Moreover, it is suggested that research with longitudinal design and comprehensive indicators be undertaken in the future.

## 1. Introduction

Environment is an external factor directly related to human activities. The influence of environment quality on people’s mental health has reached a consensus [1,2], and is widely regarded as an important predictor of mental health [3,4,5].

Academics generally have believed that mental illness not only affects social harmony and stability, but also consumes a large number of healthy lives, which has become one of the main health challenges [6]. At present, depression seems to have become one of the most common mental disorders and presents a trend of deterioration year by year [7], which seriously threatens the quality of life. Researchers have not only focused on the impacts of psychological and social factors on public depression [8], but also have begun to pay more attention to the impacts of environmental factors. With the further improvement of informatization and modernization, environmental quality and human mental health are closely intertwined. Environmental quality refers to the degree of environmental qualification, that is the suitability of the overall or some elements of the environment for human and social and economic development in a specific environment. The overall environmental quality refers to the overall quality of the environment within the scope of the national domain, and the living environment quality refers to the overall quality of the environment within the scope of the individual living in it. The perception of environmental quality can be measured by respondents’ cognitive evaluation of environmental quality (e.g., cognitive evaluation of national overall environmental quality and cognitive evaluation of the environmental quality of living community) [9].

As an important medium carrier, environment directly or indirectly induces a variety of physical and mental diseases. Long-term exposure to a polluted environment may affect people’s brain structure, lead to greater psychological pressure, then negatively affect the emotional and cognitive abilities [10], and even induce depression [11]. Although there may be a gap between environmental pollution and the public’s actual feelings, the public’s feelings on environmental pollution can also indirectly reflect the situation of environmental pollution.

Climate change [12], air pollution [13], risk perception of living in polluted areas and natural disasters [14], noise pollution perception of residential areas [15], outdoor lighting at night [16], and regional socio-economic inequality [17] have made the public worried or uncertain about the future risks, threaten the public’s emotional health, seriously damaged the public’s mental health, and can lead to depression. However, the public’s trust on the external environment is self-centered and based on the objective environment. The public’s perception of the environment determines their psychological security. People with higher psychological security may experience more self-confidence and freedom, while people with lower psychological security might undergo anxiety and even depression [18]. These show that the public’s perception of environmental quality can induce a passive mentality [19,20], and especially the negative effects of depression should be paid more attention to. Therefore, we propose the following hypothesis:

**Hypothesis** **1.** *People with a worse perception of environmental quality are facing more serious depression*.

**Hypothesis** **1a.** *People with a worse perception of overall environmental quality are facing more serious depression*.

**Hypothesis** **1b.** *People with a worse perception of living environmental quality are facing more serious depression*.

Subjective social class refers to people’s relative economic environment, which requires participants to judge how their living standards compare with other people in their city [21], including those with social power and social prestige, which have a significant impact on negative emotions and can strongly predict individual mental health [22]. Evidence indicates that an individual’s social class is closely related to internalization and externalization of behavior problems [23]. People tend to think that the lower class groups have lower expectations for the future. Researchers have found that adolescents’ expectations are negatively correlated with social class, school environment perception, and interpersonal environment perception, which directly affect their sense of security, happiness and belonging [24]. The outcome of a child’s development depends on the environment and cultural background of the family and neighborhood [25]. The incidence of depression was significantly higher in rural children, ethnic minority children, children from poor families, and children whose parents were depressed [26]. In addition, migrant workers or rural labor force, women, the divorced and widowed, low-income people, low-educated people, workers without medical insurance, and other low-level socially vulnerable groups are also more likely to have depression symptoms [27]. However, the lower social class tends to live in less developed areas, with lower education levels, lower perception of life quality, lower mental health, and higher loneliness and depression [28]. In addition, the risk perception of natural disasters and noise pollution perception in residential areas seriously affect the emotion and mental health of people living in polluted areas [14,15]. It can be seen that there are class differences in negative emotions [29], medical resources [30], stigmatization degree of mental health diagnosis [31], education level, perception of life quality, and mental health level [28]. Thus, the following hypothesis is proposed:

**Hypothesis** **2.** *People with a worse perception of environmental quality are in a lower subjective social class*.

**Hypothesis** **2a.** *People with a worse perception of overall environmental quality are in a lower subjective social class*.

**Hypothesis** **2b.** *People with a worse perception of living environmental quality are in a lower subjective social class*.

How people view their social status has been a predictive signal of mental health, and depression is closely related to cognition [32]. Socioeconomic status has brought about individual differences in cognitive function [33]. The heterogeneity of an individual’s cognitive abilities leads to differences in coping styles so that individuals show different levels of mental health in the face of pressure. Stress response rumination and negative cognitive style are strong predictors of depression [34]. Individuals with negative cognitive style tend to attribute negative life events to the common causes of lasting stability and are more likely to make further negative self-worth hints [35]. Subjective social status has a causal relationship with depressive thinking [36] and can predict health outcomes better than objective social status indicators. In addition, social status, residence, and social life stress could promote cognitive aging [37], while poverty experience and persistent cognitive impairment [38], subjective perception of health [39], and subjective perception of family social status [40] might induce an individual’s depression. It is gratifying that the improvement of cognitive function can effectively reduce the risk of depression and enhance the fluid intelligence and language understanding ability [41], because people with high cognitive ability could accurately judge problems and choose the best coping strategies so as to restore positive mental health [42]. It is worth noting that the impact of socio-economic disadvantage is similar among all races and nationalities, and the impact on health was largely driven by the high social class [43]. The advantage of socioeconomic status has significantly reduced depressive symptoms and delayed cognitive decline. There is no doubt that cognitive ability has a positive mediating effect, and higher subjective social class could effectively improve mental health [44]. Living near the park has been positively correlated with residents’ life satisfaction, which can significantly buffer the residents’ depression symptoms [45]. In order to explore the impact of environment on individuals’ depression, this study aims to introduce a new perspective to explain the impact mechanism of environmental quality perception on depression. Specifically, using the data from the 2016 China family tracking survey and regression estimation method, we empirically studied the mediating effect of subjective social class between environmental quality perception and depression. It has been found that the perception of environmental quality affects public’s subjective social class, and then changes the scope and intensity of their social comparison. Individuals with a poor perception of environmental quality may be more dissatisfied with their social class and more likely prone to be depressed. Hence, we propose the following hypothesis:

**Hypothesis** **3.** *Subjective social class plays a mediating role between environmental quality perception and depression*.

**Hypothesis** **3a.** *Subjective social class plays a mediating role between overall environmental quality perception and depression*.

**Hypothesis** **3b.** *Subjective social class plays a mediating role between living environmental quality perception and depression*.

## 2. Materials and Methods

### 2.1. Data and Sample

The data we used in this study were from the 2016 China Family Panel Studies (CFPS) conducted by the Institute of Social Science Survey (ISSS) of Peking University [46]. The China Family Panel Studies started in 2010 and is a national, comprehensive, and continuous academic survey project in China. Since 2010, the ISSS has conducted a continuous cross-sectional survey of 16,000 households covering 25 provinces, municipalities, and autonomous regions in China, which has comprehensively collected data on multiple levels, including society, economy, population, education, health, social relations, cognition, attitude, etc., and basically reflects the overall picture of China’s family economic and social life and well represents the whole country. In order to ensure the validity, authenticity, representativeness, and accuracy of the data, CFPS 2016 adopted objective sampling, such as a probability proportionate to size sampling (PPS) design, to track and collect data on multiple levels, including society, community, family, and individual levels.

Considering the complexity and comprehensiveness of environmental quality perception and subjective social class, this study only looked at survey data from 33,296 individuals aged 16 and above. The study yielded a final effective sample of 5246 after deleting invalid samples, such as “refused to answer”, “unanswerable”, and “singular value”. Data are available through the Institute of Social Science Survey Data Archive website.

As shown in Table 1, there were 5246 valid samples, including 2506 males (47.8%) and 2740 females (52.2%). Their mean age was 47.80 (SD = 16.75). Of the total 5246 respondents, 1385 owned urban household registration (26.4%) and 3861 owned rural household registration (73.6%). Further, 4207 respondents reported being married (80.2%) and 1039 unmarried (19.8%). With regard to their health condition, 3405 reported being healthy (64.9%) and 1841 unhealthy (35.1%).

### 2.2. Variables and Instrument

Environmental quality perception (EQP) was a core independent variable, which was measured by rating scale. Due to the comprehensiveness and complexity of environmental pollution, the pollution level of a certain pollutant was not enough to represent the environmental pollution level. Therefore, this study used people’s perception of environmental quality to represent the level of environmental pollution, and further discussed the impact of environmental quality on people’s mental health. Previous studies had measured environmental quality perception (EQP) from two dimensions, the cognitive evaluation of overall environmental quality perception in China (OEQP) and the cognitive evaluation of living environmental quality perception in the community (LEQP) [9]. A question (“How serious are the environmental problems in China?”) was selected, which reflected the overall environmental quality perception of independent variables in this study. Respondents could rate the extent of the answer from “not serious” to “very serious”, which assigned a value from 0 to 10, successively. A question (“How serious are the environmental problems around your community, and whether there is noise pollution, garbage stacking, etc.?”) reflected the living environmental quality perception of independent variables in this study. Respondents could rate the extent of the answer from “very good” to “very bad”, which assigned a value from 1 to 5, successively. A higher score represented worse perception of environmental quality.

Depression was a dependent variable, which was measured by the Center for Epidemiologic Studies depression scale (CES-D). The scale had been widely used as an effective method to measure individual depression, and contained 20 items, including 16 questions to measure negative feelings (such as “I feel lonely”) and 4 questions to measure positive feelings (such as “I am confident in the future”). Respondents could answer “little or no (no more than 1 day)”, “not much (1–2 days)”, “sometimes half a day (3–4 days)” and “most of the time (5–7 days)”. The responses of negative emotion items were assigned as 1,2,3 and 4, while the response assignment of positive emotion items was scored reversely. The total CES-D score was from 0 to 80 and a higher score represented more serious depression. The Cronbach’s alpha coefficient for the scale was 0.858 during this study.

Subjective social class (SSC) was a mediating variable that was measured by Likert’s 5-point rating scale. A question (“How high is your social status in the local community?”) was used to reflect subjective social class in this study. The range of options that respondents needed to answer were from very high to very low, with values ranging from 1 to 5. A higher score represented lower subjective social class.

Based on the availability of data and drawing on the need for existing research, extra variables were controlled that could affect public depression, including age, gender, household registration, health condition, and marital status. To facilitate data statistics and analysis, in terms of gender, “female” was assigned a value of 0 and “male” a value of 1. In terms of age, the values of 0, 1, and 2 were separately assigned to age groups 16–44, 45–59, and >60, respectively. For household registration, “city-registered residence” was assigned a value of 0 and “rural-registered residence” a value of 1. For health condition, “normal” and “unhealthy” were assigned a value of 0 and “relatively healthy”, “very healthy”, and “very healthy” a value of 1. In terms of marital status, we assigned “unmarried”, “cohabitation”, “divorced”, and “widowed” a value of 0 and “in marriage” a value of 1.

### 2.3. Statistical Analysis

Before statistical analysis, the data were screened according to the principle of three standard deviations above or below the mean scores. Missing values were excluded from the analysis. Descriptive statistics and correlation analyses were performed with SPSS (Version 25.0, IBM Corp, Armonk, NY, USA). We used stepwise linear regression analysis to test the mediating effect of subjective social class with maximum likelihood estimators and 95% bias-corrected confidence intervals (CIs) using 5000 bootstrapped samples repeatedly.

Specifically, the SPSS macro PROCSS plug-in was used in the inspection and methods, which was developed by Hayes [47]. We chose Model 4 to test the mediating effect of subjective social class between environmental quality perception and public depression on the basis of controlling individual social demographic and personal characteristics such as age, gender, household registration, health condition, and marital status.

## 3. Results

### 3.1. Preliminary Analysis

The descriptive statistics and correlation coefficients for the study variables are presented in Table 2. Correlation analyses indicated that depression was significantly positively correlated with subjective social class (r = 0.075, *p* < 0.01) and living environmental quality perception (r = 0.106, *p* < 0.01), while depression was negatively correlated with overall environmental quality perception (r = −0.056, *p* < 0.01). Moreover, subjective social class was significantly positively associated with environmental quality perception (r = 0.096, *p* < 0.01), overall environmental quality perception (r = 0.076, *p* < 0.01), and living environmental quality perception (r = 0.088, *p* < 0.01). In addition, environmental quality perception was significantly positively associated with overall environmental quality perception (r = 0.952, *p* < 0.01) and living environmental quality perception (r = 0.454, *p* < 0.01). These results provided good preliminary support for the hypotheses.

### 3.2. Testing the Study Mode

This study showed that environmental quality perception had a significant positive effect on public’s depression. On this basis, we further explored the mediating role of subjective social class between environmental quality perception and depression (as shown in Table 3).

Firstly, the effect of environmental quality perception on individual depression was tested by the model. The results of model 1 show that EQP had a significant positive predictive effect on depression (β = 0.095, *p* < 0.01), indicating that worse EQP probably caused more serious depression, which supported H1. As was shown in model 4, OEQP had no predictive effect on depression (β = 0.001, *p* > 0.05), indicating that OEQP had no effect on individual depression. Therefore, H1a was not proved correct. However, model 7 show that LEQP had a significant positive predictive effect on depression (β = 0.912, *p* < 0.001), which illustrated worse LEQP portended more serious depression. Therefore, H1b was verified and reflected an individual’s depression was mainly affected by LEQP, and better LEQP signified better mental health.

Secondly, the model tested the influence of EQP on individual SSC. The results of model 2 show that EQP had a significant positive prediction on SSC (β = 0.025, *p* < 0.001), suggesting that worse EQP indicated lower SSC. Therefore, H2 was asserted. Subsequently, the two dimensions of EQP were tested. The results of model 5 show that OEQP had a significant positive prediction on SSC (β = 0.020, *p* < 0.001), implying that worse OEQP appeared to lower SSC. Thus, H2a was also supported. The results of model 8 show that LEQP had a significant positive prediction on SSC (β = 0.082, *p* < 0.001), indicating that worse LEQP signified lower SSC. So, H2b was also confirmed. This indicated that SSC was affected by both OEQP and LEQP. In other words, individuals with better OEQP or LEQP owned better mental health.

In terms of the mediating effect, model 3 found that SSC played a significant mediating role in the relationship between EQP and depression (β = 0.013, *p* < 0.001, 95%CI = [0.006, 0.022]). Similarly, the results of model 9 indicated that the effect of LEQP on depression was also mediated by SSC (β = 0.041, *p* < 0.001, 95% CI = (0.019, 0.069)). Hence, both H3 and H3b were supported. However, H3a had not been confirmed by model 6.

**Figure 1:**
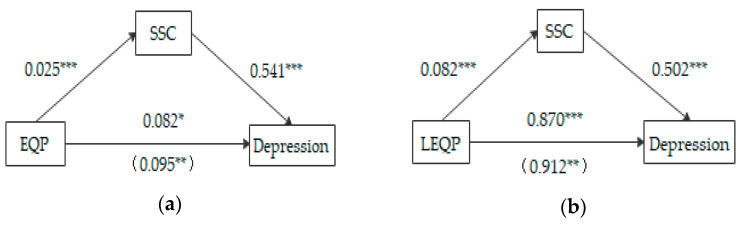
Mediation model of subjective social class. Total effect statistics in parentheses; * *p* < 0.05, ** *p* < 0.01, ***p < 0.001. (**a**) The mediating role of SSC between EQP and depression; (**b**) the mediating role of SSC between LEQP and depression.

Moreover, we adopted the bootstrapping procedure with 5,000 sub-samples to further examine the main and mediating effects. If the 95% confidence interval (CI) did not contain zero, this meant the effects were significant. Table 4 shows the analysis results. In particular, EQP was positively associated with depression (β = 0.095, SE = 0.037, 95% CI = (0.023, 0.168), CI did not include zero), and H3 was tested again. Similarly, LEQP was positively associated with depression (β = 0.912, SE = 0.114, 95% CI = (0.688, 1.136), CI also did not include zero), and these findings provided initial support for H3b. However, OEQP was not associated with depression (due to CI including zero). Hence, H3a failed and was not verified. The standardized estimates for the structural model are shown in Figure 1.

## 4. Discussion

Using national representative data, the current study attempted to contribute to the literature by investigating the impact of environmental quality perception on public depression and exploring the mediating role of subjective social class. The results show that environmental quality perception affects public’s depression through the mediating role of subjective social class. Our research established a mediation model and drew four valuable findings. First, environmental quality perception has a significant positive impact on public depression. That is to say, the worse one’s perception of environmental quality, the more serious the public depression. Second, environmental quality perception has a significant positive impact on subjective social class, which means the worse one’s perception of environmental quality, the lower the public subjective social class. Third, subjective social class has a significant positive impact on public depression, that is, the lower one’s subjective social class, the more serious the public depression. Fourth, subjective social class plays a mediating role in the relationship between environmental quality perception and public depression. This study is conducive to better understand the relationship among environmental quality perception, subjective social class, and public depression.

A large number of studies have shown the impact of environment on public depression [48,49]. Some researchers believed that environmental quality might be one of the most decisive social factors affecting an individual’s mental health and life expectancy [50]. The social and economic level of living environment affects people’s cognitive level [51]. Consistent with previous studies, this study also revealed the significant impact of environmental quality perception on public depression.

When people are in social behavior and socio-economic disadvantage, they are more vulnerable to bad environmental quality, and thus exacerbated the negative emotions [52]. For example, people with higher professional status enjoy more work autonomy, engage in less physical labor, and have less opportunities to be exposed to the risk of a poor-quality environment [52]. Similarly, higher income is usually associated with better quality of life, housing conditions, and living environment, thus improving an individual’s subjective social class perception [53]. There is no doubt that the optimization of environmental quality can improve an individual’s depression and psychological function [54]. Therefore, worse environmental perception may be associated with worse depression. However, our H1a that people with a worse perception of overall environmental quality are facing more serious depression was not supported. It is worth noting that H1b that worse perception of living environmental quality results in more serious depression, has been verified. This shows that the public’s perception of environmental quality is based on cognitive evaluation of close physical space. A more convincing conclusion may be that the public’s perception of environmental risk is based on the distance and harm degree of environmental risk. Another possible explanation for our unsubstantiated hypothesis is that people with a higher subjective social class are in a relatively better environment and have more opportunities to improve their environment or have the ability to choose a better environment, which may weaken the negative impact of environmental quality perception on depression. However, this conjecture needs further study.

Another finding of this study shows that subjective social class has a significant impact on the public’s depression, which is consistent with the existing research. A large number of studies have shown that the correlation between subjective social class and depression is related through a variety of psychological effects. They are mainly reflected in the following aspects: well-being (e.g., subjective well-being and health assessment) [22], life satisfaction [55], security (e.g., self-esteem and control [56], response to threats, and cognitive function [57,58]), sense of fairness (e.g., sense of class discrimination and stigma [59], emotion, and justice perception) [60], sense of acquisition (e.g., cultural expression and practice model [61], expected educational level [62], medical expenditures [63]), social trust (e.g., social adaptation and interpersonal trust [21]), social support (e.g., social connection [64], social support and sense of control [65], social relationship quality [66], etc.). Our results also show that people with lower subjective social class have more severe depression, which means subjective social class is significantly associated with depression. Therefore, the results further prove that higher subjective social class can effectively relieve the public’s depression.

The mediating effect model supports our hypothesis that subjective social class mediates the relationship between environmental quality perception and depression. There is adequate evidence showing that environmental quality perception and subjective social class have a significant impact on public depression, but its mechanism is lacking sufficient explanation. Social psychologists generally believed that the perception of environmental quality was the far-end factor of public depression, while the subjective social class was the near-end factor. However, early studies mainly focused on the proximal factors and ignored the distal factors [55,67]. Nevertheless, the influence of social factors on people’s depression cannot be ignored, because people’s subjective social class is largely determined by their perception of the environmental and social comparison [68]. According to the dynamic model of the theory of relative deprivation, subjective social class is the sense of deprivation when people compared their situation with a certain standard or reference and find that they are at a disadvantage, which produces negative emotions accompanied by feelings of anger and resentment [69]. The results of this study are largely consistent with the model. When people compare their environmental quality with the other relatively high-quality environments, poorer environmental quality can easily lead to a sense of deprivation, which can reduce people’s subjective perception of their own social class and induce negative depression. People with lower perception of environmental quality are less likely to report good emotions and mental health. Meanwhile, people with higher subjective social class may report better mental health. More importantly, subjective social class may mediate the relationship between environmental quality perception and depression. In other words, environmental quality perception has a direct impact on depression through subjective social class. Subjective social class reflects personal social cognition, which can be described as positive or negative. People with higher perception of environmental quality may own stronger motivation of maintaining a higher subjective social class and obtain more abundant available resources, which further shows the comparative advantage of mental health.

The negative effects of environmental quality on mental health are persistent and stable [70]. Negative environmental perception affects the public’s mental health and even leads to depression [71]. The imbalance of living environment seriously affects the social interaction between people and the surrounding environment, which influences the public’s perception of social justice [72] and negative evaluation of life well-being [73], and then affects an individual’s social emotions, leading to mental health problems [74]. Furthermore, the present paper emphasizes the important influence of subjective social class on depression and has a certain value in building a systematic evidence base for interventions and policies to reduce health inequalities. Improving the natural environment and social environment system [75,76] and enhancing people’s positive evaluation of environmental perception could help reduce class inequality and promote the development of people’s mental health and psychological function [77].

This research has potential theoretical and practical implications. On the one hand, this study provides preliminary empirical evidence from a Chinese social context and finds that there are differences in living environmental factors in mental health inequality. On the other hand, most of the existing studies only involve the impact of environmental pollution on mental health and do not explore the internal mechanism in detail. By emphasizing the correlation between environmental quality perception and depression, to some extent, this study expanded the social significance of subjective social class. Furthermore, this paper had a certain value in building a systematic evidence base for interventions and policies to reduce mental health inequalities. This study highlighted the important impact of subjective social class on mental health. Therefore, a strategy embedding intervention of subjective social class perception in public mental health promotion programs could be adopted to reduce the risk of depression.

Some limitations of the present study should be noted. Although we used the mediation model to test the relationship between variables, it was still difficult to draw accurate causal conclusions due to the cross-sectional nature of the research data. Besides, if longitudinal study data were used and multiple cognitive indicators were adopted, the results would be more convincing.

## 5. Conclusions

This study examined the impact of environmental quality perception on depression and focused on the mediating role of subjective social class. The findings indicated that depression is significantly positively associated with living environmental quality perception and negatively associated with overall environmental quality perception. Meanwhile, subjective social class significantly positively impacts depression. Furthermore, the mediating effect of subjective social class between living environmental quality perception and depression is significant. Unfortunately, the mediating effect of subjective social class between overall environmental quality perception and depression is not valid.

These findings enhanced our understanding of the relationship and mediating mechanism between environmental quality perception, subjective social class, and depression, showing that poor living environmental quality perception reduces an individual’s subjective social class and then leads to depression. It can be seen that individual depression was affected by the living environmental quality perception and subjective social class. We strongly recommend that the national environmental protection department and civil affairs department strengthen the improvement of people’s living environment so as to enhance individuals’ subjective social class and reduce the risk of depression. Moreover, it is quite necessary to carry out longitudinal design and comprehensive index research in the future.

## Figures and Tables

**Table 1:** Descriptive statistics of samples (N = 5246).

Variables	Percentage	Variables	Percentage
Age		Health condition	
Age (16–44) = 0	2137 (40.7%)	Very healthy = 1	670 (12.8%)
Age (45–59) = 1	1650 (31.5%)	Healthier = 2	946 (18.0%)
Age (above 60) = 2	1459 (27.8%)	Healthy = 3	1789 (34.1%)
Gender		General healthy = 4	1004 (19.1%)
Female = 0	2740 (52.2%)	Unhealthy = 5	837 (16.0%)
Male = 1	2506 (47.8%)	Marital status	
Household registration		Unmarried = 1	596 (11.4%)
Urban = 0	1385 (26.4%)	Married = 2	4207 (80.2%)
Rural = 1	3861 (73.6%)	Cohabitation = 3	14 (0.3%)
		Divorce = 4	98 (1.9%)
		Widowed = 5	331 (6.3%)

**Table 2:** Descriptive statistics and correlation coefficients for the study variables.

	EQP	OEQP	LEQP	SSC	D
Environmental quality perception(EQP)	-				
Overall environmental quality perception(OEQP)	0.952 **	-			
Living environmental quality perception(LEQP)	0.454 **	0.160 **	-		
Subjective social class(SSC)	0.096 **	0.076 **	0.088 **	-	
Depression(D)	−0.018	−0.056 **	0.106 **	0.075 **	-
M	9.072	6.299	2.772	2.781	32.315
SD	2.948	2.661	0.913	1.056	8.039

N = 5246, ** *p* < 0.01.

**Table 3:** Test results of mediating effects.

Variables	Model 1	Model 2	Model 3	Model 4	Model 5	Model 6	Model 7	Model 8	Model 9
Control Variables									
Age	0.530 *** (0.138)	−0.128 *** (0.019)	0.599 *** (0.138)	0.438 *** (0.138)	−0.135 *** (0.019)	0.513 *** (0.138)	0.559 *** (0.133)	−0.141 *** (0.019)	0.629 *** (0.134)
Gender	−1.210 *** (0.209)	0.059 * (0.029)	−1.242 *** (0.209)	−1.191 *** (0.209)	0.061 * (0.029)	−1.225 *** (0.209)	−1.243 *** (0.208)	0.059 * (0.029)	−1.273 *** (0.208)
Household registration	2.386 *** (0.238)	−0.121 *** (0.033)	2.452 *** (0.237)	2.314 *** (0.238)	−0.126 *** (0.033)	2.384 *** (0.237)	2.395 *** (0.235)	−0.132 *** (0.033)	2.462 *** (0.235)
Health condition	−4.959 *** (0.228)	−0.182 *** (0.032)	−4.860 *** (0.228)	−4.953 *** (0.228)	−0.185 *** (0.032)	−4.851 *** (0.228)	−4.807 *** (0.228)	−0.167 *** (0.032)	−4.723 *** (0.228)
Marital status	−1.637 *** (0.263)	−0.064 (0.036)	−1.603 *** (0.262)	−1.662 *** (0.263)	−0.066 (0.036)	−1.625 *** (0.262)	−1.648 *** (0.261)	−0.069 (0.036)	−1.613 *** (0.260)
Independent variables									
EQP	0.095 ** (0.037)	0.025 *** (0.005)	0.082 * (0.037)						
OEQP				0.001 (0.041)	0.020 *** (0.006)	−0.010 (0.041)			
LEQP							0.912 *** (0.114)	0.082 *** (0.016)	0.870 *** (0.114)
Mediator variables									
SSC			0.541 *** (0.099)			0.557 *** (0.099)			0.502 *** (0.099)
R2	0.126	0.024	0.131	0.125	0.022	0.130	0.135	0.024	0.139
F	125.449 ***	21.194 ***	112.371 ***	124.180 ***	19.339 ***	111.569 ***	136.315 ***	21.820 ***	121.094 ***

N = 5246, * *p* < 0.05, ** *p* < 0.01, *** *p* < 0.001.

**Table 4:** Non-standardized mediation analysis results.

Model Paths	Estimate	SE	BC 95% CI	
			Lower	Upper
Model paths1				
Total effect				
EQP→depression	0.095	0.037	0.023	0.168
Direct effect				
EQP→SSC	0.025	0.005	0.014	0.035
SSC→depression	0.541	0.099	0.347	0.736
EQP→depression	0.082	0.037	0.010	0.154
Indirect effect				
EQP→SSC→depression	0.013	0.004	0.006	0.022
Model paths2				
Total effect				
LEQP→depression	0.912	0.114	0.688	1.136
Direct effect				
LEQP→SSC	0.082	0.016	0.051	0.113
SSC→depression	0.502	0.099	0.309	0.696
LEQP→depression	0.870	0.114	0.647	1.094
Indirect effect				
LEQP→SSC→depression	0.041	0.013	0.019	0.069

BC, biased corrected (5000 bootstrapping sample). Control variables (age, gender, household registration, health condition, and marital status) were added to the non-standardized mediation analysis.

## Data Availability

Data are available through the Institute of Social Science Survey (ISSS), Peking University, available online website: http://www.isss.pku.edu.cn/.
